# Effects of oral fluid in post-anesthesia care unit under ultrasound monitoring on postoperative recovery quality in patients undergoing laparoscopic surgery: a randomized controlled trial

**DOI:** 10.3389/fmed.2026.1739071

**Published:** 2026-03-18

**Authors:** Mingtao Liu, Xin Zhang, Xin Liu, Yanfang Feng, Ting Li, Jiamin Bao

**Affiliations:** 1 Department of Anesthesiology and Surgery, People’s Hospital of Ningxia Hui Autonomous Region, Ningxia Medical University, Yinchuan, Ningxia, China; 2 Department of Functional Medicine, People’s Hospital of Ningxia Hui Autonomous Region, Ningxia Medical University, Yinchuan, Ningxia, China

**Keywords:** general anesthesia, laparoscopic surgery, oral rehydration, point-of-care ultrasound, postoperative nausea and vomiting, postoperative recovery quality

## Abstract

**Objective:**

To explore the impact of early oral rehydration under gastric ultrasound monitoring on the postoperative recovery quality of patients undergoing laparoscopic surgery under general anesthesia.

**Methods:**

A randomized controlled trial was conducted, dividing 94 patients who underwent laparoscopic surgery under general anesthesia into an intervention group (early oral rehydration under ultrasound guidance, *n* = 47) and a control group (traditional fasting and no drinking, *n* = 47). The primary outcome measures included the Quality of Anesthesia Recovery, postoperative thirst degree, and the time to first flatus. Secondary outcome measures included the incidence of postoperative nausea and vomiting (PONV), and postoperative hospital stay. Safety measures included the cross-sectional area and volume of the antrum, and the occurrence of aspiration, reflux, and aspiration pneumonia.

**Results:**

The QoR-15 score was higher in the intervention group than in the control group (136.0 vs. 114.0, *p* < 0.001). The thirst NRS scores at 2 h, 6 h, and 24 h postoperatively were significantly lower in the intervention group than in the control group (*p* < 0.001). The recovery time of gastrointestinal function in the intervention group was 4 h shorter than that in the control group (median time: 9.10 h vs. 13.10 h, *p* < 0.001). The incidence of PONV was reduced by 21.27% (10.64% vs. 31.91%, *p* = 0.011). The median postoperative hospital stay in the intervention group was shortened by 1 day (3.0 days vs. 4.0 days, *p* < 0.001). Ultrasound monitoring technology revealed significant improvements in the cross-sectional area and volume of the antrum, but both remained below the safety threshold of 1.5 mL/kg. No complications such as aspiration, reflux, or aspiration pneumonia were observed throughout the observation period.

**Conclusion:**

Early oral rehydration under gastric ultrasound monitoring can safely and effectively improve the postoperative recovery quality of patients undergoing laparoscopic surgery under general anesthesia, relieve thirst, promote gastrointestinal function recovery, reduce the incidence of PONV, and shorten hospital stay. It is a perioperative management strategy with clinical promotion value.

**Clinical trial number:**

This study was registered with the Chinese Clinical Trial Registry https://www.chictr.org.cn/bin/project/edit?pid=264130; ChiCTR2500099155.

## 1 Introduction

Laparoscopic surgery under general anesthesia has become a routine procedure in modern surgical practice. However, postoperative patients often face a series of recovery challenges, affecting their overall rehabilitation quality. Traditional perioperative management strategies, such as postoperative fasting, although considered safe to some extent, overlook the relief of subjective discomforts such as postoperative thirst. This neglect may delay the recovery of gastrointestinal (GI) function, increase the incidence of postoperative nausea and vomiting (PONV), and subsequently prolong hospital stays, leading to higher medical costs. The inhibitory effects of general anesthesia on gastrointestinal motility (1), combined with the traditional practice of prolonged postoperative fasting, make patients highly susceptible to Postoperative Thirst Syndrome (POTS) (incidence exceeding 70%) (2), which not only increases postoperative discomfort (3) but also delays early activities, feeding, and other recovery processes (4). Early postoperative oral fluid intake for laparoscopic patients under general anesthesia may alleviate discomfort and promote the recovery of gastrointestinal function, but it carries the risk of iatrogenic aspiration (5, 6).

In recent years, the concept of Enhanced Recovery After Surgery (ERAS) has been introduced, emphasizing multidisciplinary collaboration to optimize perioperative management, reduce surgical stress, minimize complications, and accelerate postoperative recovery. Measures include shortening postoperative fasting and drinking times. In this context, early oral rehydration, as an emerging intervention, has gradually attracted clinical attention. However, traditional fluid management strategies often rely on fixed time standards, lacking accurate assessments of individual gastric emptying function or other postoperative recovery indicators. Research in this area is limited, and existing results have inherent limitations (6).

Point-of-Care Ultrasound (POCUS) technology, as a convenient, non-invasive real-time assessment tool, has been widely applied in various clinical fields, particularly in anesthesia and critical care. By assessing the cross-sectional area of the gastric antrum and gastric volume in real-time, POCUS can help devise personalized fluid supplementation plans, maximizing both safety and efficacy (7, 8).

This study utilized ultrasound monitoring in the Postanesthesia Care Unit (PACU) to administer appropriate amounts of clear fluids orally based on the patient’s weight. Using POCUS to assess gastric volume, we observed the alleviation of thirst, the time to gastrointestinal function recovery, the incidence of PONV, and the impact on overall recovery quality 1 day post-surgery, evaluating the clinical value of ultrasound-guided early oral rehydration.

## 2 Materials and methods

### 2.1 General information

This prospective randomized controlled trial included 94 patients who underwent elective laparoscopic surgery under general anesthesia at Ningxia Hui Autonomous Region People’s Hospital from April 2025 to August 2025 and were admitted to the PACU. Patients were randomly assigned into two groups using a random number table: the control group (traditional postoperative fasting group, *n* = 47) and the intervention group (ultrasound-guided early drinking group, *n* = 47). This study was approved by the Ethics Committee of Ningxia Hui Autonomous Region People’s Hospital (Approval No.: 2025-WJW-023) and registered with the Chinese Clinical Trial Registry (Identifier: ChiCTR2500099155). Informed consent was obtained from all participants. The study design, patient enrollment, randomization, allocation, follow-up, and analysis were conducted in accordance with the CONSORT guidelines. During the study period, postoperative oral intake management for laparoscopic procedures at our institution was based on a fixed 6-h fasting protocol rather than routine early oral feeding.

#### 2.1.1 Inclusion criteria

Patients undergoing laparoscopic surgery under general anesthesia and admitted to the PACU, with a surgery duration ≤ 2 h, American Society of Anesthesiologists (ASA) classification I–III, aged 18–65 years, and body mass index (BMI) ≤ 35 kg/m^2^. All participants were conscious adults and provided written informed consent for study participation.

#### 2.1.2 Exclusion criteria

Patients with postoperative fluid restrictions; those with impaired pharyngeal or esophageal function; gastroesophageal reflux disease; respiratory tract obstruction; other conditions preventing fluid intake; BMI > 35 kg/m^2^. Patients with respiratory tract obstruction or a known or predicted difficult airway prior to surgery were excluded due to the potential risk of delayed recovery of airway protective reflexes and increased aspiration risk during early postoperative oral hydration.

#### 2.1.3 Randomization and blinding

Randomization was performed using a computer-generated random number table, with the random sequence securely maintained by independent statisticians. The study adhered to a double-blind design with allocation concealment, achieved through the use of sealed envelopes. Both the study participants (patients and their families) and the researchers (including those involved in patient visits and data analysis) were blinded to the group assignments. Following randomization, group allocation information was encoded by independent personnel, ensuring confidentiality throughout the intervention and data collection phases. Nurses administering the interventions were also blinded, and subjective outcome measures, including QoR-15 scores and PONV incidence, were assessed by blinded evaluators, who were unaware of the group allocations.

### 2.2 Study methods

#### 2.2.1 Control group

Patients in the control group followed the standard postoperative fasting protocol of our institution, which requires fasting for 6 h after laparoscopic surgery under general anesthesia, followed by gradual reintroduction of oral fluids according to patient tolerance. After the 6-h fasting period, patients first received 50 mL of room-temperature sterile water. If tolerated (no nausea, vomiting, coughing, or signs of aspiration within 30 min), the volume was increased to 100 mL every 2 h for the next 6 h (i.e., up to 12 h post-operatively). Thereafter, patients were allowed to drink ad libitum according to their thirst and preference, provided they continued to tolerate oral intake without adverse events. This protocol was applied uniformly to all control-group participants to ensure consistency while respecting individual tolerance and preference. This fasting duration reflects routine clinical practice at our center during the study period and was adopted to ensure patient safety and provide a consistent comparator for evaluating the effects of ultrasound-guided early oral hydration. Although oral fluid intake in the control group was permitted after 6 h postoperatively, the volume was initially limited and progressed gradually based on patient tolerance, which might be insufficient to promptly alleviate subjective thirst.

#### 2.2.2 Intervention group

After monitoring vital signs in the PACU, ultrasound was used to assess the gastric antrum cross-sectional area. The patient was placed in a semi-recumbent position (head elevated 30°) and given 1 mL/kg of sterile water. The patient was observed for 1 h; if no adverse reactions (coughing, nausea, vomiting) occurred, they were transferred back to the ward. The postoperative protocol after 6 h was the same as the control group. The volume of 1 mL/kg was selected based on gastric ultrasound–derived aspiration risk thresholds reported in previous studies, indicating that a gastric volume < 1.5 mL/kg is associated with a low risk of pulmonary aspiration. Administering 1 mL/kg of clear fluid was therefore considered as a conservative and safe initial volume, being sufficient to relieve postoperative thirst while maintaining gastric volume well below the aspiration risk threshold. Using a weight-based dosing strategy also minimized inter-individual variability and allowed individualized hydration while ensuring consistency across participants.

#### 2.2.3 Ultrasound assessment and measurement

The patient was placed in the right lateral position. A Huasheng ultrasound machine (Latat SE) with a convex array probe (2–5 MHz) was used to locate the gastric antrum. The scan was performed in the sagittal plane below the xiphoid process, identifying the “bullseye” sign. The anteroposterior (AP) and craniocaudal (CC) diameters were measured and gastric antrum area calculating (formula: CSA = *Π* × AP × CC/4) then the gastric volume (formula: *V* = 27 + 14.6 × CSA-1.28 × age) (7). A gastric volume <1.5 mL/kg indicated that the patient could safely drink fluids. Gastric antrum area and volume were reassessed at 30 and 60 min post-fluid intake. If the gastric volume exceeded 1.5 mL/kg, the patient was excluded and monitored. All procedures were performed by qualified intermediate-level physicians with standardized internal training. Measurements were independently repeated three times by two physicians, and the average value was used.

#### 2.2.4 Blood oxygen monitoring

In the PACU, SpO₂ was continuously monitored using a Masimo pulse oximeter. SpO₂ at 30 min post-fluid intake was recorded for the intervention group, and SpO₂ at the same interval for the control group.

#### 2.2.5 Continuous monitoring and safety

Respiratory Monitoring: SpO₂ and respiratory status were monitored during and after surgery, especially in the PACU and ward. If oxygen saturation decreased or airway instability occurred, intubation or adjustment of oxygen therapy was performed. Systematic respiratory and gastric emptying monitoring was conducted postoperatively to ensure patient safety. Although oral fluid was administered only when patients were fully conscious with intact airway protective reflexes, coughing, aspiration, and pulmonary complications were monitored as safety endpoints to ensure comprehensive postoperative surveillance. Residual anesthetic effects, opioid use, or unexpected changes in mental status may still pose a theoretical aspiration risk in the immediate postoperative period. Monitoring these events could therefore confirm the safety of the intervention rather than to imply a high expected incidence.

Aspiration and Pulmonary Complications: If hypoxia, coughing, or abnormal pulmonary imaging (e.g., chest X-ray showing aspiration) occurred, the hospital’s rapid response protocol was activated, including pulmonary physiotherapy, antibiotics, and follow-up imaging if necessary.

Safety Screening for Enrollment: Strict inclusion criteria were followed, excluding patients with severe gastrointestinal motility disorders or respiratory diseases to reduce the risk of postoperative aspiration.

#### 2.2.6 Perioperative anesthesia, analgesia, and antiemetic management

All patients received standardized general anesthesia according to institutional protocols, including anesthetic induction, maintenance, and postoperative analgesia. Prophylactic antiemetic therapy was routinely administered pre- and/or intraoperatively based on institutional practice and individual risk assessment.

### 2.3 Outcome measures

#### 2.3.1 Primary outcomes

① Anesthesia recovery quality: Assessed using the 15-item QoR-15 scale (8, 9) 24 h post-surgery, with a total score range of 0–150, covering pain, psychological support, and self-care ability. ② Thirst level: Evaluated using the Numerical Rating Scale (NRS) (10), with a range of 0–10, where 0 represents no thirst and 10 represents extreme thirst. ③ Time to first flatus: The time from the end of surgery to the patient’s first anal flatus, measured in hours.

#### 2.3.2 Secondary outcomes

① PONV incidence: Observed for nausea and vomiting. PONV was evaluated as a comparative postoperative outcome between groups and was not intended to establish a direct causal relationship with the intervention alone. ② Postoperative hospital stay: Total days from surgery to discharge (criteria for discharge: normal body temperature, ability to eat independently, no need for intravenous analgesia). ③ SpO₂ measurements: Recorded as per 1.2.4.

#### 2.3.3 Safety outcomes

① Gastric antrum area and volume: Recorded at 30 and 60 min post-PACU admission.

② Severe adverse events: Documented for both groups, including aspiration, reflux, and aspiration pneumonia, which were monitored as safety endpoints. Gastrointestinal adverse effects, including postoperative ileus and vomiting, were assessed through time to first flatus (primary outcome) and PONV incidence (secondary outcome).

### 2.4 Statistical methods

#### 2.4.1 Sample size calculation

Sample size estimation was conducted based on the primary outcomes: postoperative thirst NRS score and time to first flatus. Previous studies reported a mean difference of 2.5 ± 1.8 points in thirst scores and a reduction of 3.5 ± 2.1 h in time to first flatus between the intervention and control groups (11, 12). Using a two-tailed *α* of 0.05 and 80% power (*β* = 0.20), sample size calculations performed with PASS 2023 software indicated that 41 participants per group were required for the thirst score (minimum clinically important difference ≥ 2 points, combined standard deviation 1.9) and 37 participants per group for time to first flatus (reduction ≥ 3 h, combined standard deviation 2.2). Given the larger sample size requirement for the thirst score, it was selected as the primary endpoint. Accounting for a 10% dropout rate, a total of 94 participants (47 per group) were enrolled.

#### 2.4.2 Statistical analysis

Statistical analysis was performed using SPSS 26.0 software (IBM, Armonk, NY, United States). Continuous data were presented as mean ± standard deviation (*^−^x* ± *s*), with inter-group comparisons conducted using independent-samples *t*-tests. For abnormally distributed data, the median (M) and interquartile range (IQR) were reported, and differences between groups were assessed using the Mann–Whitney *U* test and *z*-score conversion. Categorical data were presented as frequency (%) and were compared using the *χ*^2^ test or the Fisher’s exact test, as appropriate. Statistical significance was defined as *p* < 0.05.

## 3 Results

### 3.1 General information

A total of 116 patients were screened for eligibility for laparoscopic surgery, with 22 excluded (10 due to high BMI, 5 due to postoperative fluid restrictions, and 7 who refused participation). Ultimately, 94 patients were included in the study. There were no statistically significant differences between the two groups in terms of gender, BMI, surgery type, surgery time, anesthesia time, or postoperative fasting time (*p* > 0.05), as shown in Table 1.

**Table 1:** Comparison of general information between two groups.

Item	Control group (*n* = 47)	Intervention group (*n* = 47)	*t*/*χ*^2^/*Z*	*P*-value
Gender [*n* (%)]				
Female	26 (55.32)	22 (46.81)		0.409
Male	21 (44.68)	25 (53.19)		0.409
BMI (*x̄* ± *s*)/(kg/m^2^)	24.30 ± 3.21	25.60 ± 4.10	−1.459	0.148
Surgery type [*n* (%)]				0.167
Cholecystectomy	31 (65.96)	37 (78.72)		
Appendectomy	16 (34.04)	10 (21.28)		
Surgery time M(IQR)/(min)	55.0 (24.0)	54.0 (10.0)	−0.413	0.679
Anesthesia time M(IQR)/(min)	114.0 (30.0)	114.0 (30.0)	−0.717	0.474
Fasting time M(IQR)/(h)	12 (2.7)	13 (4.5)	−1.918	0.055
Water restriction time M(IQR)/(h)	10 (4.0)	9.7 (1.2)	−0.333	0.739

No statistically significant differences (*P* > 0.05).

### 3.2 Comparison of postoperative recovery indicators

The intervention group showed significant improvement in postoperative recovery quality, with a higher QoR-15 total score than the control group (*p* < 0.05). Gastric function recovery in the intervention group was significantly better than in the control group, with a first flatus time of 9.10 (3.30) hours, 4.0 h shorter than the control group’s 13.10 (2.00) hours, a statistically significant difference (*p* < 0.001). The PONV incidence in the intervention group was 10.64% (5/47), significantly lower than the control group’s 31.91% (15/47), with a statistically significant difference (*p* < 0.05). The median postoperative hospital stay in the intervention group was 1 day shorter than the control group, with a statistically significant difference (*p* < 0.05). No significant difference was found in SpO₂ between the two groups, as shown in Table 2.

**Table 2:** Postoperative recovery and QoR-15 score comparison between control and intervention groups.

Indicator/dimension	Control group (*n* = 47)	Intervention group (*n* = 47)	Test statistic (*t*/*χ*^2^/*Z*)	*P*
QoR-15 score (points)	114.0 (11.00)	136.0 (8.00)	−7.510	<0.001
First flatus time (hours)	13.10 (2.00)	9.10 (3.30)	−5.854	<0.001
PONV (*n*, %)	15 (31.91%)	5 (10.64%)	2.520	0.011
Hospital stay time (days)	4.0 (1.0)	3.0 (1.0)	−3.987	<0.001
SpO₂ (%)	93.45 (12.3)	95.79 (10.2)	0.219	0.445
Thirst NRS scores (PACU admission)	8.0 (1.0)	9.0 (1.0)	0.174	–
Thirst NRS scores (PACU discharge)	9.0 (1.0)	2.0 (2.0)	<0.001	–
Thirst NRS scores (2 h post-op)	9.0 (1.0)	2.0 (3.0)	<0.001	–
Thirst NRS scores (6 h post-op)	8.0 (1.2)	0.0 (1.0)	<0.001	–
Thirst NRS scores (24 h post-op)	8.0 (2.0)	0.0 (1.0)	<0.001	–
Physical comfort (QoR-15)	31.5 (10.4)	35.3 (11.2)	−4.12	<0.001
Emotional state (QoR-15)	26.8 (9.3)	30.4 (10.1)	−3.50	<0.001
Physical independence (QoR-15)	11.7 (4.0)	13.0 (4.5)	−2.30	0.024
Psychological support (QoR-15)	12.3 (4.1)	14.5 (4.3)	−2.83	0.006
Pain (QoR-15)	12.2 (3.9)	13.0 (4.2)	−2.10	0.039

For Postoperative Recovery Indicators, statistical significance is indicated by *P* < 0.05.

For Thirst NRS Scores, significant differences are indicated by *p* < 0.0125.

Values in parentheses represent standard deviations (SD) or interquartile ranges (IQR) as noted.

-, none.

### 3.3 Comparison of thirst level scores

The intervention group had significantly lower thirst NRS scores at PACU discharge, 2, 6, and 24 h post-surgery compared to the control group, with statistically significant differences (*p* < 0.05), as shown in Table 2. Despite initiation of oral fluid intake at 6 h postoperatively in the control group, thirst scores remained elevated at 24 h, indicating that delayed and limited fluid intake did not adequately relieve postoperative thirst.

### 3.4 QoR-15 dimension scores

In all five dimensions, the intervention group had higher scores than the control group, particularly in physical comfort, emotional state, and psychological support, with statistically significant differences (*p* < 0.05), as shown in Table 2.

### 3.5 PONV incidence by time level

Before *p*-value adjustment, there were significant differences in vomiting upon PACU discharge, nausea at 2 h post-surgery, vomiting at 2 h post-surgery, and nausea at 6 h post-surgery. After adjustment, all differences were not statistically significant. As shown in Table 3.

**Table 3:** PONV incidence at different time points.

Time point	Frequency (incidence)	*χ*^2^ value	*P*-value	Degrees of freedom
Nausea at PACU entry	43 (0.46)	10.97	0.1009	3
Vomiting at PACU entry	22 (0.23)	1.48	0.2232	3
Nausea at PACU discharge	16 (0.17)	22.64	0.2015	3
Vomiting at PACU discharge	6 (0.06)	10.04	0.0182	3
Nausea at 2 h post-op	11 (0.12)	6.83	0.0275	3
Vomiting at 2 h post-op	8 (0.09)	5.28	0.0423	3
Nausea at 6 h post-op	7 (0.07)	3.91	0.0335	3
Vomiting at 6 h post-op	3 (0.03)	2.52	0.0681	3
Nausea at 24 h post-op	9 (0.10)	4.88	0.1807	3
Vomiting at 24 h post-op	5 (0.05)	1.76	0.7856	3

### 3.6 Comparison of gastric antrum cross-sectional area and gastric volume

At 30 and 60 min post-intervention, the gastric antrum cross-sectional area and volume were significantly increased in the intervention group. Compared with the control group, these differences were statistically significant (*p* < 0.001). The gastric volume remained below the established aspiration risk threshold of <1.5 mL/kg, as recommended by multiple authoritative studies (11–13), as shown in Table 4.

**Table 4:** Gastric antrum cross-sectional area and gastric volume at two time points post-intervention.

Group	Control group (*n* = 47)	Intervention group (*n* = 47)	*t*-value	*P*-value
Gastric area (m^2^)				
30 min	4.24 (2.61)	7.07 (1.89)*	−6.424	< 0.001
60 min	4.12 (2.45)	5.91 (1.28)*	−4.333	< 0.001
Gastric volume (ml/kg)				
30 min	0.37 (0.66)	0.99 (0.11)*	−7.189	< 0.001
60 min	0.36 (0.69)	0.71 (0.19)*	−4.522	< 0.001

*Compared to the control group, *p* < 0.05, statistically significant differences. *M*(SD) represents mean and standard deviation.

### 3.7 Primary and secondary endpoint testing methods

The normality of each variable was assessed, and appropriate statistical tests were applied. Multiple comparison adjustments, handling of missing data, and the calculation of confidence intervals for consistency tests are detailed. These procedures are outlined in Supplementary Table 1.

### 3.8 Adverse events

No adverse events, including aspiration, reflux, or aspiration pneumonia, occurred in either group throughout the study. A detailed list is provided in Supplementary Table 2.

## 4 Discussion

This randomized controlled trial integrated bedside gastric ultrasound monitoring with ERAS principles to evaluate the effects of ultrasound-guided early oral hydration on postoperative recovery in patients undergoing elective laparoscopic surgery under general anesthesia. The findings demonstrate that early, individualized oral hydration based on gastric ultrasound assessment significantly improves overall postoperative recovery quality, reduces thirst severity, accelerates gastrointestinal function recovery, decreases the incidence of PONV, and shortens postoperative hospital stay, without increasing aspiration-related adverse events.

It was revealed that the intervention group achieved significantly higher QoR-15 total and domain scores compared with the control group. The QoR-15 is a validated, multidimensional tool reflecting physical comfort, emotional state, physical independence, psychological support, and pain. The observed 22-point improvement in the intervention group exceeds the reported minimal clinically important difference, indicating a meaningful enhancement in patient-perceived recovery quality. This improvement likely reflects the combined effects of timely hydration, improved comfort, reduced stress response, and earlier restoration of physiological function rather than a single isolated mechanism. Early postoperative hydration may improve microcirculatory perfusion and attenuate postoperative inflammatory responses, thereby reducing systemic discomfort and fatigue (14–16). In addition, earlier gastrointestinal recovery—evidenced by a significantly shorter time to first flatus—facilitates earlier nutritional intake and functional recovery. Previous studies have demonstrated that early oral intake stimulates enteric nervous system activity, enhances splanchnic blood flow, and promotes intestinal motility, thereby reducing the risk of postoperative ileus (17, 18). These findings are consistent with reports that early postoperative feeding activates the gut–brain axis and supports recovery after abdominal surgery (19).

The intervention group experienced a median reduction of one postoperative hospital day compared with the control group. Importantly, this difference occurred in the absence of postoperative complications, suggesting that improved physiological recovery and symptom control facilitated earlier readiness for discharge. Within the ERAS framework, early restoration of oral intake is a cornerstone intervention that contributes to reduced morbidity, faster mobilization, and improved patient satisfaction (20). From a health-economic perspective, even modest reductions in hospital stay can decrease healthcare costs and improve bed utilization, particularly in high-volume surgical centers.

Postoperative thirst is one of the most common and distressing symptoms reported by surgical patients, with a prevalence exceeding 70% after general anesthesia (21). In this study, thirst NRS scores were significantly lower in the intervention group at PACU discharge and at all postoperative time points up to 24 h. These findings highlight the effectiveness of early oral hydration in relieving thirst, beyond the effect of delayed or limited fluid intake. Prolonged preoperative fasting, intraoperative fluid loss, and anesthetic-related suppression of salivary secretion contribute to postoperative hypovolemia and oral dryness (22). Endotracheal intubation further exacerbates pharyngeal and oral mucosal irritation, intensifying thirst perception. Even small volumes of oral fluid can moisten the oral mucosa, improve subjective comfort, and rapidly reduce thirst intensity. Persistent thirst has been associated with anxiety, irritability, impaired concentration, and increased risk of postoperative delirium, which contradicts the principles of patient-centered and comfort-oriented care emphasized in ERAS pathways (16, 23, 24). Therefore, effective thirst management represents an important yet often underestimated component of postoperative recovery.

The intervention group demonstrated a lower overall incidence of PONV compared with the control group. It should be emphasized that anesthesia management, analgesic regimens, and prophylactic antiemetic strategies were standardized between groups; therefore, PONV was analyzed as a comparative postoperative outcome rather than a direct causal effect of hydration alone. Nevertheless, early correction of hypovolemia may reduce nausea by improving tissue perfusion and attenuating vagal and chemoreceptor-mediated emetic reflexes (25). Additionally, fluid supplementation may reduce postoperative hyperosmolar states and mitigate serotonin-mediated pathways involved in PONV pathophysiology (26). Symptom relief from thirst and discomfort may also reduce neuroendocrine stress responses, further decreasing susceptibility to nausea important vomiting (3, 27). These findings support the concept that optimized perioperative fluid management plays an important adjunctive role in PONV prevention within ERAS programs.

Traditional postoperative fasting protocols rely on fixed time intervals and do not account for interindividual variability in gastric emptying. The key innovation of this study lies in the use of real-time bedside gastric ultrasound to guide individualized early oral hydration. Gastric ultrasound has been validated as a reliable, noninvasive tool for assessing gastric content and aspiration risk (17, 28). In this study, gastric antrum cross-sectional area and volume increased gradually after oral hydration in the intervention group, reflecting recovery of gastric motility. Importantly, gastric volume remained consistently below the accepted aspiration risk threshold of 1.5 mL/kg (11–13), and no cases of reflux, aspiration, or aspiration pneumonia occurred. This approach optimizes the balance between safety and clinical benefit, aligning with contemporary perioperative risk-stratification strategies (29, 30). The portability and ease of use of point-of-care ultrasound further support its feasibility for routine clinical application across diverse healthcare settings.

This study has several important limitations that should be acknowledged. Firstly, this was a single-center study with a relatively small sample size, which might limit the generalizability of the findings and introduce potential selection bias. Secondly, although gastric ultrasound is a validated bedside tool, its accuracy may be affected by postoperative abdominal distension, residual pneumoperitoneum, and gastrointestinal gas following laparoscopic surgery, which could influence gastric volume estimation. Thirdly, some outcome measures, including QoR-15 scores, thirst NRS scores, and PONV assessment, were subjective and might be influenced by individual perception, emotional state, and cultural factors, despite the use of blinding procedures. Fourthly, this study did not perform subgroup analysis in high-risk populations (e.g., elderly, diabetic, or obese patients), nor did it compare different fluid types, volumes, or cost-effectiveness of hydration strategies. Fifthly, patients with known or predicted difficult airways were excluded for safety reasons; therefore, the applicability of ultrasound-guided early oral hydration in this population requires further investigation. Finally, long-term outcomes, including 30-day postoperative complications, readmission rates, and long-term functional recovery, were not evaluated. These limitations indicate the need for larger, multicenter studies with extended follow-up in the future research.

In conclusion, ultrasound-guided early oral hydration with a small volume of clear fluid (1 mL/kg) is a safe and effective postoperative strategy. It significantly improves recovery quality, alleviates thirst, accelerates gastrointestinal function recovery, reduces PONV incidence, and shortens hospital stay in patients undergoing laparoscopic surgery under general anesthesia. This individualized approach represents a practical and clinically valuable enhancement to ERAS-based perioperative care.

## Data Availability

The original contributions presented in the study are included in the article/Supplementary material, further inquiries can be directed to the corresponding authors.
