# Supplementary material for: Effects of oral fluid in post-anesthesia care unit under ultrasound monitoring on postoperative recovery quality in patients undergoing laparoscopic surgery: a randomized controlled trial
Source: Front Med (Lausanne). 2026 Mar 18;13:1739071. doi: 10.3389/fmed.2026.1739071 (PMC13038860; doi:10.3389/fmed.2026.1739071)
Supplement: Supplementary file 1 [file Table_1.docx]

**Supplementary Table 1 Testing Methods for Primary and Secondary Endpoints.**

| **Indicator** | **Normality (Shapiro-Wilk Test)** | **Test Method** | **Multiple Comparison (P-value Correction)** | **Missing Data Handling** | **Adjusted P-value** | **Consistency Check** |
| --- | --- | --- | --- | --- | --- | --- |
| QoR-15 Score | Non-normal (p < 0.05) | Mann-Whitney U | Bonferroni correction | Mean imputation | <0.05 | ICC = 0.92 (0.85, 0.96) |
| First Flatus Time | Non-normal (p < 0.05) | Mann-Whitney U | Bonferroni correction | Mean imputation | <0.05 | ICC = 0.79 (0.71, 0.84) |
| PONV (Nausea at PACU Entry) | Non-normal (p < 0.05) | Mann-Whitney U | Holm-Bonferroni | Remove missing data | <0.0125 | Kappa = 0.75 (0.63, 0.88) |
| PONV (Vomiting at PACU Entry) | Non-normal (p < 0.05) | Mann-Whitney U | Holm-Bonferroni | Remove missing data | <0.0125 | Kappa = 0.70 (0.55, 0.85) |
| PONV (Nausea at 2 Hours Post-op) | Non-normal (p < 0.05) | Mann-Whitney U | Holm-Bonferroni | Remove missing data | <0.0125 | Kappa = 0.80 (0.70, 0.90) |
| PONV (Vomiting at 2 Hours Post-op) | Non-normal (p < 0.05) | Mann-Whitney U | Holm-Bonferroni | Remove missing data | <0.0125 | Kappa = 0.78 (0.68, 0.88) |
| PONV (Nausea at 6 Hours Post-op) | Non-normal (p < 0.05) | Mann-Whitney U | Holm-Bonferroni | Remove missing data | <0.0125 | Kappa = 0.82 (0.74, 0.91) |
| PONV (Vomiting at 6 Hours Post-op) | Non-normal (p < 0.05) | Mann-Whitney U | Holm-Bonferroni | Remove missing data | <0.0125 | Kappa = 0.76 (0.65, 0.87) |
| PONV (Nausea at 24 Hours Post-op) | Non-normal (p < 0.05) | Mann-Whitney U | Holm-Bonferroni | Remove missing data | <0.0125 | Kappa = 0.74 (0.63, 0.85) |
| PONV (Vomiting at 24 Hours Post-op) | Non-normal (p < 0.05) | Mann-Whitney U | Holm-Bonferroni | Remove missing data | <0.0125 | Kappa = 0.80 (0.70, 0.90) |
| Thirst NRS Score (PACU Discharge) | Non-normal (p < 0.05) | Mann-Whitney U | Holm-Bonferroni | Remove missing data | <0.0125 | ICC = 0.85 (0.77, 0.92) |
| Thirst NRS Score (2 Hours Post-op) | Non-normal (p < 0.05) | Mann-Whitney U | Holm-Bonferroni | Remove missing data | <0.0125 | ICC = 0.88 (0.80, 0.94) |
| Thirst NRS Score (6 Hours Post-op) | Non-normal (p < 0.05) | Mann-Whitney U | Holm-Bonferroni | Remove missing data | <0.0125 | ICC = 0.90 (0.85, 0.95) |
| Thirst NRS Score (24 Hours Post-op) | Non-normal (p < 0.05) | Mann-Whitney U | Holm-Bonferroni | Remove missing data | <0.0125 | ICC = 0.92 (0.88, 0.96) |
| Gastric Antrum Area (30min) | Normal (p > 0.05) | t-test | Holm-Bonferroni | Remove missing data | <0.025 | ICC = 0.75 (0.71, 0.82) |
| Gastric Antrum Area (60min) | Normal (p > 0.05) | t-test | Holm-Bonferroni | Remove missing data | <0.025 | ICC = 0.80 (0.70, 0.84) |
| Gastric Volume (30min) | Normal (p > 0.05) | t-test | Holm-Bonferroni | Remove missing data | <0.025 | ICC = 0.77 (0.75, 0.85) |
| Gastric Volume (60min) | Normal (p > 0.05) | t-test | Holm-Bonferroni | Remove missing data | <0.025 | ICC = 0.72 (0.68, 0.86) |
| Aspiration, Reflux, Aspiration Pneumonia | None | None | None | None | None | Kappa = 0.95 (0.93, 1.00) |
