# Supplementary material for: Effects of oral fluid in post-anesthesia care unit under ultrasound monitoring on postoperative recovery quality in patients undergoing laparoscopic surgery: a randomized controlled trial
Source: Front Med (Lausanne). 2026 Mar 18;13:1739071. doi: 10.3389/fmed.2026.1739071 (PMC13038860; doi:10.3389/fmed.2026.1739071)
Supplement: Supplementary file 2 [file Table_2.docx]

**Supplementary Table 2 Detailed List of Complications and Safety Events in Both Groups.**

| **Event** | **Quantity** | **Description** | **Monitoring Method** | **Response Method** |
| --- | --- | --- | --- | --- |
| Oxygen Saturation Drop | 0 | SpO₂ <90% or sustained decline | Continuous pulse oximetry, SpO₂, blood gas analysis | Oxygen therapy |
| Respiratory Rate Drop | 0 | Respiratory rate <8 breaths/min, or arterial CO₂ >50mmHg | Respiratory rate monitoring, arterial blood gas analysis | Mechanical ventilation support |
| Airway Obstruction | 0 | Laryngeal or glottic spasm causing airway blockage | Signs of no ventilation, no airflow | Oxygen administration, intubation |
| Aspiration | 0 | Chemical or aspiration pneumonia caused by gastric or oropharyngeal contents | Chest imaging (e.g., chest X-ray, CT), clinical symptoms monitoring | Mechanical ventilation if severe |
| Pulmonary Inflammation or Pulmonary Edema | 0 | Aspiration pneumonia, pulmonary edema, or ARDS | Blood gas analysis, chest X-ray, SpO₂ monitoring | Extended hospital stay, symptomatic treatment |
| Readmission to PACU | 0 | Re-admission due to pulmonary complications (e.g., aspiration pneumonia) within 30 days post-surgery | Medical record review, hospital stay analysis, 30-day follow-up | Oxygen therapy, respiratory support |
| High Gastric Residual Volume | 0 | Gastric content residue >1.5 mL/kg, increasing aspiration risk | Gastric ultrasound measurement, residual volume assessment | Decreased intake, gastrointestinal motility agents, gastric tube drainage |
| Laparoscopic Pneumoperitoneum Residual | 0 | Residual pneumoperitoneum causing diaphragm elevation, decreased lung compliance, and thoracic function restriction | Postoperative imaging (chest X-ray, CT) or blood gas analysis | Gas release, proper positioning, oxygen supplementation, chest physiotherapy |
| Gastroesophageal Reflux | 0 | Gastric acid reflux causing esophageal irritation or aspiration risk | Clinical symptoms monitoring, gastrointestinal motility tests | Antacid medication, head-up position, fasting |
